# Revealing the role of lattice distortions in the hydrogen-induced metal-insulator transition of SmNiO_3_

**DOI:** 10.1038/s41467-019-08613-3

**Published:** 2019-02-11

**Authors:** Jikun Chen, Wei Mao, Binghui Ge, Jiaou Wang, Xinyou Ke, Vei Wang, Yiping Wang, Max Döbeli, Wentong Geng, Hiroyuki Matsuzaki, Jian Shi, Yong Jiang

**Affiliations:** 1 Beijing Advanced Innovation Center for Materials Genome Engineering, School of Materials Science and Engineering, University of Science and Technology Beijing, 100083 Beijing, China; 2 School of Engineering, the University of Tokyo, 2-11-16 Yayoi, Bunkyo-ku, Tokyo, 113-0032 Japan; 3 Beijing National Laboratory for Condensed Matter Physics, Chinese Academy of Sciences, 100190 Beijing, China; 4 Beijing Synchrotron Radiation Facility, Institute of High Energy Physics, Chinese Academy of Sciences, 100049 Beijing, China; 5 Department of Mechanical and Aerospace Engineering, Case Western Reserve University, Cleveland, OH 44106 USA; 6 Department of Applied Physics, Xi’an University of Technology, 710054 Xi’an, China; 7 Department of Materials Science and Engineering, Rensselaer Polytechnic Institute, Troy, New York, NY 12180 USA; 8 Laboratory of Ion Beam Physics, ETH Zurich, CH-8093 Zurich, Switzerland

## Abstract

The discovery of hydrogen-induced electronic phase transitions in strongly correlated materials such as rare-earth nickelates has opened up a new paradigm in regulating materials’ properties for both fundamental study and technological applications. However, the microscopic understanding of how protons and electrons behave in the phase transition is lacking, mainly due to the difficulty in the characterization of the hydrogen doping level. Here, we demonstrate the quantification and trajectory of hydrogen in strain-regulated SmNiO_3_ by using nuclear reaction analysis. Introducing 2.4% of elastic strain in SmNiO_3_ reduces the incorporated hydrogen concentration from ~10^21^ cm^−3^ to ~10^20^ cm^−3^. Unexpectedly, despite a lower hydrogen concentration, a more significant modification in resistivity is observed for tensile-strained SmNiO_3_, substantially different from the previous understanding. We argue that this transition is explained by an intermediate metastable state occurring in the transient diffusion process of hydrogen, despite the absence of hydrogen at the post-transition stage.

## Abstract (web summary)

Proton doping can induce metal-insulator transitions in rare-earth nickelates, demonstrating the complex interplay between dopants and electronic degrees of freedom. Chen et al. use results on strained films to argue that local proton-induced lattice distortions strongly influence the transition.

## Introduction

Recently, ion-induced quantum phase transitions have been demonstrated in several strongly correlated materials systems that have interesting applications in enabling artificial intelligence^1–3^, high-performance energy conversion^4^, biological sensing^5^, and multifunctional logic/memory devices^6^. In contrast to conventional semiconductors, *d-*orbital-band-correlated systems usually exhibit extremely complex electronic configurations and phase diagrams, which can be varied abruptly upon either electrostatic^5,6^ or chemical doping^1,2^. Recent discoveries were demonstrated in correlated nickelates^1,2,4,5^, SrCoO_x_^6^ and VO_2_^7^, in which either strong correlations, such as the Mott-Hubbard U, could be manipulated to enable metal-insulator transition (MIT) or electronic magnetic structures could be tuned to reach multiple magnetic ground states.

Among these ion species, the proton/hydrogen system is a particularly intriguing one due to its fast kinetics, ubiquitous presence, and mid-value electronegativity. Proton/hydrogen interactions with 3*d-*band-electron-correlated oxide materials and the resultant phase transitions, e.g., Mott-Hubbard among multiple electronic phases has brought up an exciting direction for exploring new physics and emerging multifunctional electronic devices^1–7^. However, despite these technical breakthroughs, a critical question remains unsolved: what is the fundamental microscopic mechanism leading to the correlated phase transition? Owing to their small atomic weight and high volatility, the presence of a hydrogen atom (^1^H) or proton (^1^H^+^) is rather difficult to directly quantify. In one previous investigation, the isotope of the deuterium (^2^D) isotope rather than ^1^H is used to demonstrate the physical presence of hydrogen within electrochemically hydrogenated SrCoO_3_^6^. Nevertheless, despite the similarity in chemical properties for ^1^H and ^2^D, their physical properties, such as density, diffusion kinetics, and volatility, are considerably different^8,9^. For other *d*-band-correlated systems, such as hydrogenated SmNiO_3_, although a sharp increase in the resistivity by eight orders of magnitude was observed upon proton doping^1,2^, a solid experiment-verified atomic understanding of the role of protons has not yet been presented.

In previous investigations, the hydrogen-induced highly insulating state of SmNiO_3_ was hypothetically attributed to the transformation from the electron itinerant orbital state $Ni^{3 + }t_{2g}^6e_g^1$ to the electron-localized state $Ni^{2 + }t_{2g}^6e_g^2$ owing to the hydrogen doping^1,2,4,5^. Nevertheless, this understanding omitted the role of possible subtle structural phase transition, which appear to be inevitable but beyond the resolution of most instruments. For example, it may be possible that the orbital reconfiguration of nickel is also achievable via kinetic processes during hydrogenation even without the physical presence of (or doping by) the hydrogen elements. This is seen previously from the irreversibility of hydrogen-induced defect behaviors, such as hydrogen embrittlement^10,11^, cavitation/blistering^12^, and interface failure^13^, owing to the defects that are formed or developed in the hydrogen diffusion path^10–15^. Finding the root causes of hydrogen-induced switching in correlated electron states by quantifying the hydrogen composition is critical within the field of hydrogen-induced strong correlations in condensed matter.

In this article, we demonstrate that the electron-localized insulating state observed in the hydrogenated SmNiO_3_ under tensile strain is more likely a direct consequence of a proton incorporation/removal-induced transient chemical state (a proton-induced oxygen-removed metastable state), via direct ^1^H quantification by using nuclear reaction analysis (NRA). Introducing tensile lattice distortions to single crystalline SmNiO_3_ films is shown to reduce the hydrogen incorporation concentration but elevates the hydrogen-induced electron localization and the resistivity. Near edge X-ray absorption fine structure (EXAFS) and Rutherford backscattering (RBS) analysis indicate a more distinct transition in the orbital configuration from an electron itinerant to electron-localized state is achieved in tensile-distorted SmNiO_3_ that is not related to the final hydrogen composition. The transition is instead associated with the transient chemical state formed in the transient diffusion process of hydrogen, despite the absence of hydrogen at the post-transition stage.

## Results

### Quantifying hydrogen via nuclear reaction analysis

**Fig. 1:**
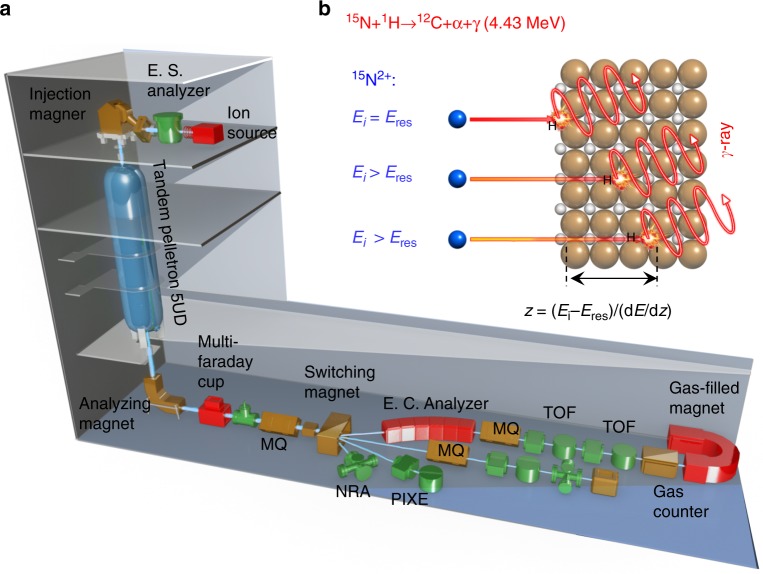
Illustration of the nuclear reaction analysis. **a** Schematic illustration of the accelerator system for the nuclear reaction analysis (NRA). The system consists of components, such as the ion source, electrostatic spherical (E.S.) analyzer, injection magnet, electrostatic cylindrical (E.C.) analyzer, several magnetic quadrupole-lens (M.Q.), time of flight (TOF), and analyzing cambers. In the present work, the system is mainly used for the NRA measurement, while the ultra high vacuum NRA, Rutherford backscattering (RBS), elastic recoil detection analysis (ERDA), and particle induced X-ray emission (PIXE) can be also performed in additional chambers and altering the ion source. **b** The working principle for quantitative detection of the hydrogen composition as a function of distribution depth by using nuclear reaction analysis (NRA). Only ^15^N^2+^ ions with kinetic energies (*E*_K_) of 6.385 MeV after penetrating SmNiO_3_ (Energy loss: 3.1945 keV nm^−^^1^) can be resonant with the hydrogen element and yield detectable gamma-rays

To date, the only way to directly quantify the absolute amount of ^1^H isotope within a solid is to use NRA, based on the nuclear resonance reactions between high-energy (MeV) ^15^N^2+^ ions from an accelerator with ^1^H-releasing detectable gamma-rays^16–18^. As illustrated in Fig. 1a, to probe the hydrogen concentration located at various depth, the hydrogenated SmNiO_3_ was irradiated by ^15^N^2+^ incident ion beams at different kinetic energies (*E*_K_). The nuclear resonance with hydrogen can only take place when the *E*_K_ of the ^15^N^2+^ after traversing in solid (*E*_K_ loss of 3.1945 keV nm^−1^ for SmNiO_3_) is within a narrow range of ~6.385 MeV^18^. By varying the *E*_K_ of the incident ^15^N^2+^ ion beam from 6.3 MeV to 7.3 MeV and measuring the respective yield of gamma-ray intensity from the resonance^16^, the concentration of hydrogen as a function of the allocation depth within SmNiO_3_ is obtained. More details of the quantification of the hydrogen concentration from the NRA spectrum are given in the Supplementary Methods.

### Strain regulation for SmNiO_3_

**Fig. 2:**
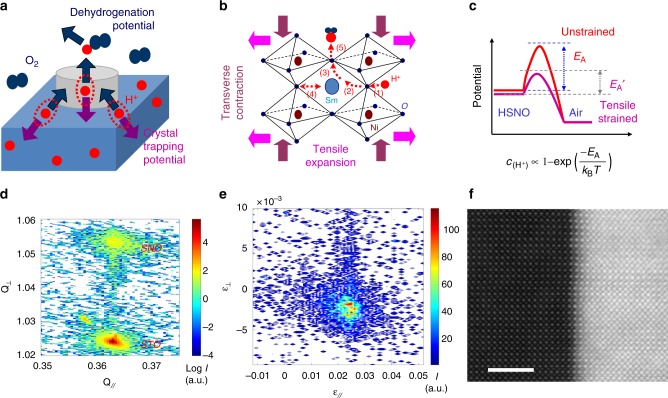
Tensile distortion of SmNiO_3_. **a** Illustration of the two competing processes taking place within the lattice of SmNiO_3_: (1) Hydrogen diffusing out of the material driven by the dehydrogenation potential when exposed in an oxygen containing atmosphere, (2) being trapped within SmNiO_3_ due to the energy barrier provided by the lattice. **b** Illustrating the proton transport processes within SmNiO_3_: (1) proton incorporation, (2) rotational diffusion and (3) transport of protons, (4) bending and stretching of the Ni-O bond in the opposite side. Imposing tensile distortion upon SmNiO_3_ improves step (2) and (3) owing to the elongated Ni-O, and thereby enhances the proton diffusion coefficient. **c** Fast diffusion of hydrogen is expected owing to the reduced energy barrier (*E*_A_) after tensile distortion of SmNiO_3_. **d** Reciprocal space mapping (RSM) of SmNiO_3_/SrTiO_3_ (001) and **e** the strain distribution of the film material converted from RSM. **f** Cs-corrected STEM high-angle annular dark-field (HAADF) image of the interface for the SmNiO_3_/SrTiO_3_ (001). Scale bar is 3 nm

Figure 2a illustrates the general concept for regulating the stabilized hydrogen incorporation concentration within the hydrogenated SmNiO_3_. More details on the electrode configuration and pattern can be found on Supplementary Figure 1. When exposing the hydrogenated SmNiO_3_ in the air, the dehydrogenation potential to drag out the hydrogen (or proton) from H_x_SmNiO_3_ via the platinum catalyst counteracts with the crystal trapping potential (the thermal dynamical and dynamical barrier for hydrogen to diffuse out of the oxide) to impede the dehydrogenation, as illustrated in Fig. 2a. The crystal trapping potential is mainly associated to the energy barrier (*E*_A_) for the incorporated hydrogen to overcome before being dragged out of the crystal by the oxygen molecule in the air. The *E*_A_ is expected to be reduced when imposing a biaxial tensile strain upon SmNiO_3_, owing to the reduced repulsion between the Ni site with the proton to improve the rotational diffusion of proton and further transfer to neighboring oxygen, as illustrated in Fig. 2b. To further demonstrate how tensile distortion affects the migration of hydrogen in SmNiO_3_, the energy migration barrier under different tension strain by using density functional theory (DFT)-based Vienna Ab initio Simulation Package was calculated. As more details in Supplementary Discussions, imparting a tensile distortion to ~2% reduces the diffusion energy barrier and increases the transition rate of hydrogen by 14 times at room temperature. Our calculation is in well agreement with the previous reports^19–21^ that an enhanced proton diffusion coefficient as previously observed in tensile-strained proton conducting oxides. Owing to the reduced *E*_A_ as illustrated in Fig. 2c for dehydrogenation, a smaller hydrogen composition is expected for more tensile-strained SmNiO_3_ via the same processes of hydrogenation followed by an exposure in air.

To introduce as-proposed biaxial lattice distortion, the single crystalline SmNiO_3_ thin films were co-lattice grown on SrTiO_3_ (001), (La,Sr)(Al,Ta)O_3_ (001), or LaAlO_3_ (001) substrates by pulsed laser deposition. The quasi-single crystal structure of as-grown SmNiO_3_ with the same orientation to the substrate is demonstrated by their X-ray diffraction (XRD) pattern as shown in Supplementary Figure 2a–f. The difference in lattice constant between the substrate and co-lattice-grown thin films imparts interfacial stains and distorts the lattice of SmNiO_3_^22–26^. The strained perovskite phase was stabilized by co-lattice deposition on the template of single crystal substrate lattice^22–24^ that reduces the formation free energy^27–29^.

As shown in Fig. 2d, e, the SmNiO_3_ exhibits a tensile distortion of ~−2.4% when co-lattice grown on SrTiO_3_ (001), noticing a smaller lattice constant (*a*_0_) of the film (*a*_0,film_ = 3.807 Å) compared to the substrate (*a*_0,substrate_ = 3.905 Å). This is confirmed by the reciprocal space mapping (RSM) as shown in Fig. 2d, where the in-plane lattice of SmNiO_3_ is locked by SrTiO_3_. Figure 2e further shows the strain distribution map along both in-plane and cross-plane directions, converted from its RSM^30^. It clearly presents the biaxial tensile distortion of the film material is demonstrated by the expansion of the in-plane lattice by ~2.4%, and the respective cross-plane transverse contraction by ~−0.3%. The preservation of tensile distortion at a relatively large magnitude of lattice mismatches may associate to the high tolerance in distorting the NiO_6_ octahedra as known for the thermodynamically instable rare-earth nickelates. In addition, the variation in kinetic processes when using different deposition approaches is also expected to influence the preservation of the tensile interfacial strain, which was preserved for using PLD^25^ and relaxed for using chemical vapor deposition^31^. Figure 2f shows the cross-section morphology of SmNiO_3_/SrTiO_3_ (001) interface, where a co-latticed interface between the single crystalline SmNiO_3_ film and the SrTiO_3_ substrate was observed. As compared to growing on SrTiO_3_, a reduced tensile distortion of ~−1.6% is expected when growing SmNiO_3_ on (La,Sr)(Al,Ta)O_3_ (001) substrate (*a*_0_ = 3.87 Å), while the SmNiO_3_ grown on LaAlO_3_ (001) substrates (*a*_0_ = 3.79 Å) is slightly compressive strained up to ~0.4%. Supplementary Figure 2g and h provides the XRD and RSM results for as-grown SmNiO_3_/(La,Sr)(Al,Ta)O_3_ (001) and SmNiO_3_/LaAlO_3_ (001). The surface morphology of as-grown SmNiO_3_ films on the three difference substrates are demonstrated in Supplementary Figure 3, which shows a reduced surface roughness with an increasing tensile distortion.

### Relating H composition to resistivity for distorted H_*x*_SmNiO_3_

**Fig. 3:**
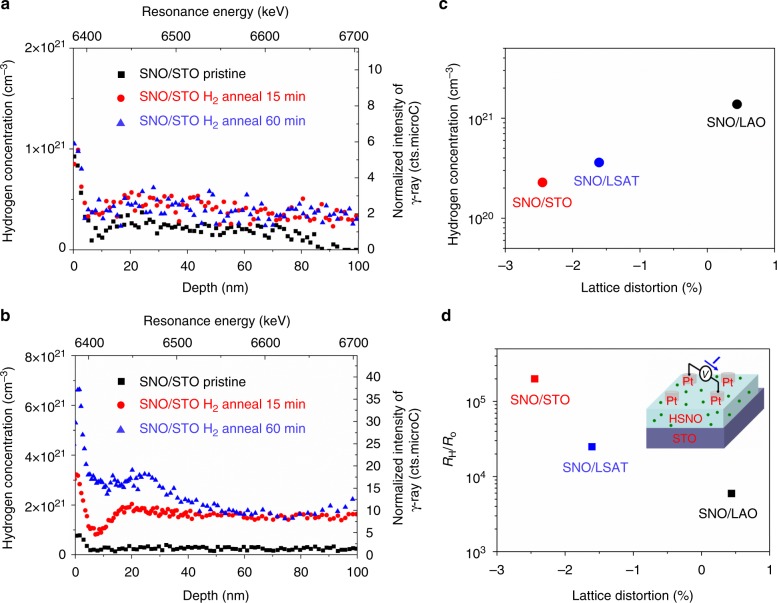
Hydrogen profiles and resistivity of H_x_SmNiO_3_ upon distortions. **a,** **b** Hydrogen depth profiles for **a** SmNiO_3_/SrTiO_3_ and **b** SmNiO_3_/LaAlO_3_ in the 1% H_2_/He atmosphere after designed annealing durations. Black square: pristine SmNiO_3_; red dot: upon hydrogen annealing for 15 min; blue triangle: upon hydrogen annealing for 60 min **c** Hydrogen concentration after annealing for 15 min and **d** the respective enhancements of the resistivity, plotted as a function of the lattice distortions for SmNiO_3_ grown on different single crystal substrates of SrTiO_3_ (red), (La,Sr)(Al,Ta)O_3_ (blue), and LaAlO_3_ (black)

The same hydrogenation process was performed for the platinum patterned SmNiO_3_ grown on different substrates similar to the previous reports. In brief, the array of dot-shaped platinum electrodes were deposited on the surface of the SmNiO_3_ thin films, and the samples were annealed in 1% H_2_/He gas at 300 °C for 15–60 min, followed by the same exposure time in the air before NRA (see more details in Supplementary Methods and Supplementary Figure 1). Figure 3a, b shows the hydrogen concentration depth profile before and after the hydrogenation measured by NRA for SmNiO_3_/SrTiO_3_ (001) and SmNiO_3_/LaAlO_3_ (001), respectively, while the ones for SmNiO_3_/(La,Sr)(Al,Ta)O_3_ (001) is shown in Supplementary Figure 4a. It is worth noticing that intensity of the surface peak in the NRA spectrum is associated to the native hydrogen (i.e., H_2_O) absorbed by the surface as well as the near surface instrumental function (see more details in Supplementary Figure 4), while the intensity in the bulk material away from the surface is proportional to the hydrogen composition^16^. The hydrogen concentration incorporated within the SmNiO_3_ film material after annealing for 15 min saturated at a concentration of 2.3 × 10^20^ cm^−3^, 3.6 × 10^20^ cm^−3^, and 1.4 × 10^21^ cm^−3^ when growing on SrTiO_3_, (La,Sr)(Al,Ta)O_3_, and LaAlO_3_, respectively. To exclude the potential proton incorporation or conduction through the SrTiO_3_ substrate, Supplementary Figure 4b shows the NRA spectrum for the hydrogenated SmNiO_3_/SrTiO_3_ sample measured at a larger depth until the interfacial region, and no effective hydrogen signal was observed in the SrTiO_3_. A reducing tendency in the incorporated hydrogen concentration when enhancing tensile lattice distortion of SmNiO_3_ was observed, as more clearly demonstrated in Fig. 3c. This is in agreement to our expectation as demonstrated previously. Nevertheless, despite incorporating a smaller hydrogen composition, a more tensile-strained SmNiO_3_ shows a more significant enhancement in the resistivity after the same hydrogenation process, as shown in Fig. 3d (the absolute resistances are shown in Supplementary Figure 5).

The above observation is in contradiction to the previous hypothesis that the metal to insulator transition was triggered by the presence of the hydrogen element within the material lattice^1,2,4,5^. As further demonstrated in Supplementary Figure 6, the resistivity of the hydrogenated SmNiO_3_ can be fully recovered to the initial magnitude via annealing in O_2_ at 300 °C for 30 min, known as a dehydrogenation procedure. Even at room temperature, the resistivity of the hydrogenated SmNiO_3_ gradually reduced by a long-time exposure in the air. However, by annealing it in the vacuum (pressure below 10^−3^ Pa) at 300 °C for 30 min, the resistivity of the hydrogenated SmNiO_3_ is further enhanced for both SmNiO_3_/SrTiO_3_ (001) as compared to SmNiO_3_/LaAlO_3_. Annealing in the vacuum will not increase the hydrogen incorporation concentration in SmNiO_3_, and this is further demonstrated by the NRA measurement where rather low hydrogen concentration was observed for the hydrogenated SmNiO_3_ sample further annealed in the vacuum.

### Transient state

**Fig. 4:**
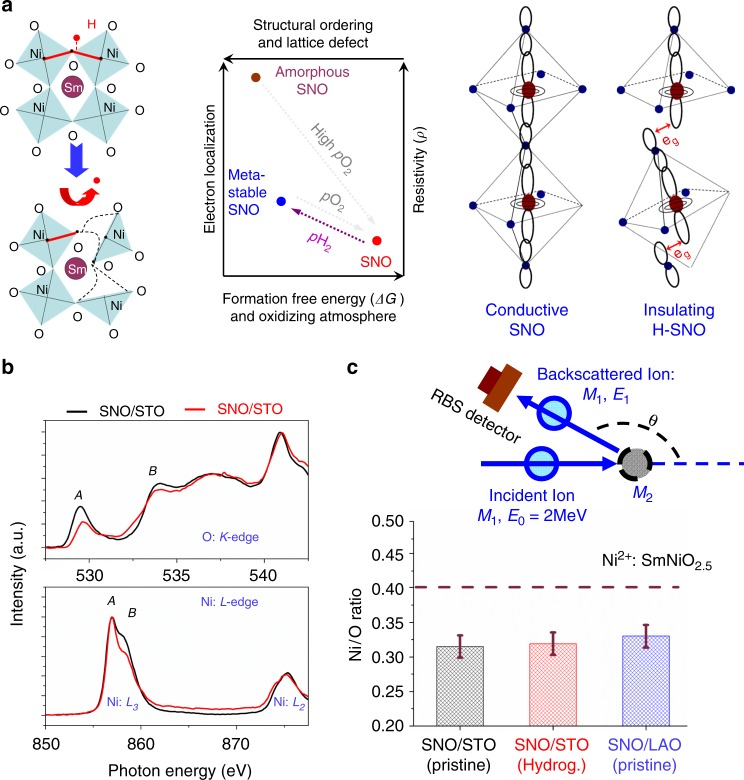
Transient state in proton regulation of SmNiO_3_. **a** Illustrating the transition from the electron itinerant state to highly electron localized state triggered by the transient process during hydrogen diffusion, instead of the physical presence of the hydrogen composition. The structural disordering owing to lattice distortions results in the opening in band gap. **b** Near edge X-ray absorption fine structure (NEXAFS) analysis for SmNiO_3_/SrTiO_3_ (red line) and SmNiO_3_/LaAlO_3_ (black line). **c** Ratios of the Ni/O composition measured for SmNiO_3_/SrTiO_3_, hydrogenated SmNiO_3_/SrTiO_3_, and SmNiO_3_/LaAlO_3_ by using the Rutherford backscattering (RBS). The upper scheme illustrates the RBS detection

Instead of the physical hydrogen doping effect, the hydrogenation process may also result in a transient chemical state occurring in the transient diffusion process of hydrogen, despite the absence of hydrogen at the post-transition stage. This is further supported by the reduced crystallinity of SmNiO_3_, as demonstrated by XRD and RSM patterns in this work (see Supplementary Figure 7 and 8), and also observed previously in ref. ^27^. It is also worth noticing that a more significant variation in RSM pattern was observed for SmNiO_3_/SrTiO_3_ compared to SmNiO_3_/LaAlO_3_, as shown in Supplementary Figure 8. As for resistivity–temperature relations in both SmNiO_3_/SrTiO_3_ and SmNiO_3_/LaAlO_3_ systems, they can be found on Supplementary Figure 9. It indicates the stretches and distortions of the lattice atoms and bonds are due to the transient diffusion process of hydrogen, and such changes cannot be recovered even by further lowering the hydrogen composition, as illustrated by Fig. 4a. This is kinetically favored since the enhanced disorder of lattice atoms elevates the system entropy and may induce a dip in the free energy of SmNiO_3_ (ΔG > 0)^27,28^. By annealing the hydrogenated SmNiO_3_ in oxygen (previously known as the dehydrogenation process), the more oxidizing atmosphere draws the chemical potential towards the formation of crystallized SmNiO_3_^28^, which may render the metastable state converting back to the ground state. Such a process is in a great analogy to crystallization of amorphous SmNiO_3_ when annealed at high oxygen pressures, as reported by refs. ^27–29^ This understanding is in consistency to the previous observations that the potential oxygen vacancies generated in oxides when annealing in vacuum can also result in huge resistance change in oxide thin films, in which case the hydrogen composition is absent^32,33^. The tensile distortion further facilitates the formation of oxygen vacancies^34,35^, which may result in more significant enhancement in the resistivity of the SmNiO_3_ as also observed in this work.

For a more in-depth investigation of the enhanced resistivity via hydrogenation observed for tensile-strained SmNiO_3_, near edge X-ray absorption fine structure (NEXAFS)^36,37^ analysis was performed for SmNiO_3_/SrTiO_3_ (001) and SmNiO_3_/LaAlO_3_. As reported previously, the orbital configuration associated to various valence states of Ni can be probed by comparing the NEXAFS spectrums of the Ni: *L*-edge and O: *K*-edge, as demonstrated in Fig. 4b. The Ni: *L*-edge usually contains two parts, while the Ni: *L*_3_ originates from the Ni 2p → Ni 3d transition, and usually splits into two peaks, reflecting the *t*^6^_2g_*e*^2^_g_ (Ni^2+^, Peak A) and *t*^6^_2g_*e*^1^_g_ (Ni^3+^, Peak B), respectively^36,38,39^. Before hydrogenation, the tensile-distorted SmNiO_3_/SrTiO_3_ shows a reduced proportion in Peak B as compared to SmNiO_3_/LaAlO_3_, indicating a reduced proportion in the *t*^6^_2g_*e*^1^_g_ orbital configurations. The further hydrogenation treatment further reduces the proportion in Peak B in Ni: *L*_3_ spectrums more significantly for SmNiO_3_/SrTiO_3_ compared to SmNiO_3_/LaAlO_3_, as shown in Supplementary Figure 10. These observations are further evidenced by a more significant reduction in the valance state of nickel for SmNiO_3_/SrTiO_3_ (001) as compared to SmNiO_3_/LaAlO_3_ demonstrated by the X-ray photoelectron spectroscopy as shown in Supplementary Figure 11.

The O: *K* spectrum exhibits a pre-peak A (~529 eV) that was attributed to the Ni:3*d*-O:2*p* hybridization (*d*^8^*L* configuration), and was previously used to monitor the oxygen vacancy formation^36,39^. The peaks appearing at the higher photon energies were expected to reflect the more oxygen depleted orbital configurations, i.e., *d*^9^*L* (Peak B)^39^. Before hydrogenation, the tensile-strained SmNiO_3_/SrTiO_3_ shows a smaller proportion of pre-peak A in O: *K*-edge, as compared to SmNiO_3_/LaAlO_3_. This is in agreement with the previous understanding that tensile distortion can result in weakened Ni-O interactions when forming the bonding orbital, by either generating oxygen vacancies or reducing the valence state of Ni. As a result, the construction of the tensile-distorted NiO_6_ octahedron is expected to be more easily destroyed by the hydrogen diffusion. Upon hydrogenation, a more significant reduction in the proportion of pre-peak A is observed in O: *K*-edge for H-SmNiO_3_/SrTiO_3_ compared to H-SmNiO_3_/LaAlO_3_ (see Supplementary Figure 10a and b).

The above observations are in agreement with our understanding that it is easier to cause deviations of the oxygen lattice atoms in the more tensile-distorted SmNiO_3_, resulting in more significant enhancement of the material resistivity. Nevertheless, the deviations of the oxygen lattice atoms do not necessarily deplete the oxygen composition and form conventional oxygen vacancies, in which case a composition of SmNiO_2.5_ is expected when Ni^3+^ is fully reduced to Ni^2+^. When measuring the thin film composition before and after hydrogenation via Rutherford backscattering (RBS), we observed no significant variations in the oxygen composition, as compared in Fig. 4c (see the RBS spectrum in Supplementary Figure 12). Therefore, the hydrogenation-induced electron localization in SmNiO_3_ is at least not simply associated to the depletion in oxygen composition to reduce nickel. In contrast, the kinetic process of hydrogenation (Supplementary Figure 13, Supplementary Table 1 and Supplementary Figure 14) is expected to form a thermodynamically more stabilized insulating state of SmNiO_3_ by driving the potential to reduce the pristine-positive formation free energy and without varying the chemical composition.

## Discussion

The present understanding also supports the previous observations associated with hydrogenated SmNiO_3_^1,2,5^ from the kinetic perspective. For example, Zuo et al demonstrated that the formation of the insulating phase of SmNiO_3_ was related to the dynamic modulation and path during the hydrogenation, which is known to be a habituation-based plasticity behavior^2^. Such kinetic dependent behavior in the hydrogen-induced phase transition may be more associated with the variety in forming the metastable chemical state when altering the hydrogenation potential, rather than varying the hydrogen doping content. Similar kinetic dependencies were also recently observed by Shi et al^1^ and Zhang et al^5^, when performing the hydrogenation of SmNiO_3_ via hydrogen annealing or electrochemical approaches, respectively. In both situations, the resistivity of the hydrogen-induced insulating phase was elevated by improving the dynamic strength, i.e., elevating the temperature or enhancing the applied external voltage. Nevertheless, the specific mechanisms associated to the enhancement in resistivities of hydrogenated VO_2_ or SrCoO_2.5_ may be different to the one for SmNiO_3_, owing to the complexity of metal-insulator transitions for various correlated oxides.

In summary, we demonstrate quantitative and direct hydrogen detection within hydrogenated single crystalline SmNiO_3_ thin films-based nuclear resonance reactions between high-energy ^15^N^2+^ and ^1^H. By regulating the lattice mismatch between the film and substrate to impart tensile distortion up to 2.4% upon SmNiO_3_, the incorporated hydrogen concentration is reduced from ~10^21^ to ~10^20^ cm^−3^. Despite the reduction of the hydrogen concentration, a more significant hydrogen-induced enhancement in resistivity was observed for the more tensile-strained SmNiO_3_. Further combined results from near edge X-ray absorption fine structure and Rutherford backscattering analysis indicate that a more significant transition in the orbital configuration from an electron itinerant state to an electron-localized one is achieved in tensile-distorted SmNiO_3_ without dependence on the hydrogen concentration. Assisted by density functional theory calculations, this transition is demonstrated to be associated with a transient chemical state occurring in the transient diffusion process of hydrogen, despite the absence of hydrogen at the post-transition stage.

## Methods

### Sample preparation

Thin SmNiO_3_ thin films were grown on SrTiO_3_ (001), LaAlO_3_ (001), and (La,Sr)(Al,Ta)O_3_ (001) single crystal substrates by pulsed laser deposition (PLD) at 20 Pa *p*O_2_ and a substrate temperature of 650 °C. For the hydrogenation treatment, the platinum electrodes were patterned on the surface of SmNiO_3_, followed by annealing in 1% H_2_/He gas at 300 °C for 15–60 min. More detailed descriptions associated for the sample preparation are presented in the Supplementary Methods.

### Sample characterization

High-angle annular dark-field (HAADF) and annular bright-field (ABF) scanning transmission electron microscopy (STEM) experimental techniques were carried out on JEM-ARM 200 F. The crystal structures were characterized by X-ray diffraction (XRD) and reciprocal space mapping (RSM). The diffraction patterns of [114] reciprocal space vectors from the film and substrate were projected at [110] and [001], representing the in-plane and cross-plane reciprocal space vector, respectively. The resistance of as-grown thin films was measured in vacuum by using a commercialized CTA-system within the temperature range from 300–550 K. The oxygen composition of the thin film was measured by Rutherford backscattering (RBS) in ETH Zurich by using a 2 MeV ^4^He beam and a silicon PIN diode detector at θ = 168°. The collected RBS data were simulated using the RUMP software, and the uncertainty for the as-measured composition is within 5%. The nuclear reaction analysis (NRA) measurement was performed in the Micro Analysis Laboratory, Tandem accelerator (MALT) at The University of Tokyo. More detailed descriptions with the sample characterization are given in the Supplementary Methods.

## Supplementary information

Supplementary information

## Data Availability

The data that support the findings of this study are available from the corresponding authors upon reasonable request.
