# Supplementary material for: Revealing the role of lattice distortions in the hydrogen-induced metal-insulator transition of SmNiO3
Source: Nat Commun. 2019 Feb 11;10:694. doi: 10.1038/s41467-019-08613-3 (PMC6370778; doi:10.1038/s41467-019-08613-3)
Supplement: Supplementary file 1 — Supplementary information [file 41467_2019_8613_MOESM1_ESM.pdf]

Supplementary Information

Revealing the role of lattice distortions in the hydrogen-induced metal-insulator transition of $SmNiO_3$

Jikun Chen[1†], Wei Mao[2†], Binghui Ge[3], Jiaou Wang[4], Xinyou Ke[5], Vei Wang[6], Yiping Wang[7],Max Döbeli[8], Wentong Geng[1], Hiroyuki Matsuzaki[2], Jian Shi[7]* and Yong Jiang[1]**

[1]Beijing Advanced Innovation Center for Materials Genome Engineering, School of Materials Science and Engineering, University of Science and Technology Beijing, Beijing 100083, China
[2]School of Engineering, The University of Tokyo, 2-11-16 Yayoi, Bunkyo-ku, Tokyo 113-0032, Japan
[3]Beijing National Laboratory for Condensed Matter Physics, Chinese Academy of Sciences, Beijing 100190, China
[4]Beijing Synchrotron Radiation Facility, Institute of High Energy Physics, Chinese Academy of Sciences, Beijing 100049, China
[5]Department of Mechanical and Aerospace Engineering, Case Western Reserve University, Cleveland, Ohio 44106, United States
[6]Department of Applied Physics, Xi'an University of Technology, Xi'an 710054, China
[7]Department of Materials Science and Engineering, Rensselaer Polytechnic Institute, Troy, New York 12180, United States
[8]Laboratory of Ion Beam Physics, ETH Zurich, CH-8093 Zurich, Switzerland

Correspondence: Prof. Jikun Chen (jikunchen@ustb.edu.cn), Prof. Jian Shi (shij4@rpi.edu), and Prof. Yong Jiang (yjiang@ustb.edu.cn).

[†]J. Chen and W. Mao contributed equally to this work.

Supplementary Methods

Thin $SmNiO_3$ thin films were grown on $SrTiO_3$ (001), $LaAlO_3$ (001) and (La,Sr)(Al,Ta)O_3 (001) single crystal substrates by pulsed laser deposition (PLD) by using ceramic targets with nominal compositions of $SmNiO_3$. The 248 nm laser at a constant ablation fluence of around 1.8 J.cm^2 was used to ablate the target material at an oxygen pressure of around 20 Pa within a vacuum chamber with the repetition frequency of 10 Hz. During the deposition, the substrates were heated to 650 °C, and the deposition thickness of the films were around 200 nm. High-angle annular dark-field (HAADF) and annular bright-field (ABF) scanning transmission electron m microscopy (STEM) experimental techniques were carried out on JEM-ARM 200F TEM operated at 200 kV with a cold field emission gun and aberration correctors for both probe-forming and imaging lenses. The crystal structures were characterized by X-ray diffraction (XRD) and reciprocal space mapping (RSM). The diffraction patterns of [114] reciprocal space vectors from the film and substrate were projected at [110] and [001], representing the in-plane and cross-plane reciprocal space vector ($Q_{//}$ and $Q_{\perp}$), respectively. The RSM result from [114] diffraction pattern demonstrates the in-plane lock between film and substrate in the other in-plane direction of [100]. The resistance of as-grown thin films was measured in vacuum by using a commercialized CTA-system within the temperature range from 300 K- 550 K.

To hydrogenate as-grown $SmNiO_3$ thin films, the patterns of platinum electrode (round shape and with diameter of 50 μm, see scheme S1) were deposited on the surface of the film material. The samples were annealed in 1% H_2/He gas at 300 °C for 15-60 minutes, followed by the same exposure time in the air before NRA. The dehydrogenation was performed by annealing the samples in oxygen at 300 °C for 30 minutes.

The oxygen composition of the thin film was measured by Rutherford Back Scattering (RBS) by using a 2 MeV ^{4}He beam and a silicon PIN diode detector at θ = 168°. The collected RBS data were simulated using the RUMP software. The uncertainty for as-measured composition is within 5%.

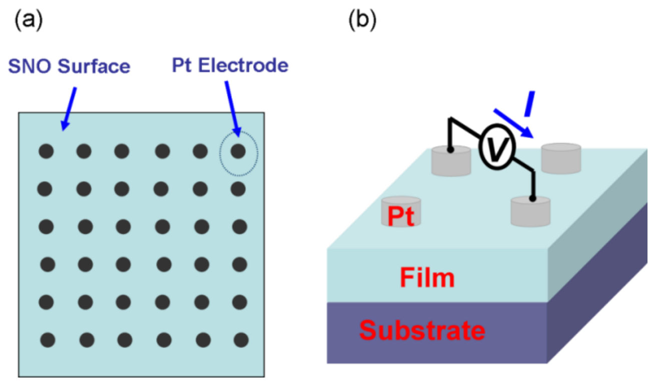

Supplementary Figure 1. Illustrating the shape of Pt pattern and the *I-V* measurements. **(a)** Illustrating the shape and spacing of the round shaped Pt electrode (diameter of 50 micrometer) deposited on the surface of $SmNiO_3$. **(b)** Illustrating the measurement of resistance before and after the hydrogenation process.

The nuclear reaction analysis (NRA) measurement was performed in the Micro Analysis Laboratory, Tandem accelerator (MALT) at The University of Tokyo. Owing to the resonant nature of the nuclear reaction, only the $^{15}N^{2+}$ ions with kinetic energies (E_K) of 6.385 MeV can resonant with the hydrogen element and yield detectable gamma-ray yield. Otherwise, at an E_K level of MeV range that much higher than the ones associated to the outer or inner shells of electrons, the kinetic loss is mainly related to the elastic scatterings by the atoms at a constant rate per penetration depth. Taking the present $SmNiO_3$ as an example, the constant of proportionality is the reciprocal of the energy loss cross section (3.1945 keV nm^{-1}) that can be considered as a constant (within 1 %) for thin films. The energy loss cross section (3.1945 keV nm^{-1}) of $SmNiO_3$ is derived based on supplementary ref. [20] and [1]. In brief, the stopping power (S) for the compound $SmNiO_3$ can be calculated by using the Bragg`s Rule, which is the composition-weighed average of elemental stopping powers for Sm, Ni and O: S_{SmNiO3}= (N_{Sm}×M_{Sm}×S_{Sm}+ N_{Ni}× M_{Ni}×S_{Ni}+ N_O×M_O×S_O) ×ρ(N_{Sm}×M_{Sm}+ N_{Ni}× M_{Ni}+ N_O×M_O)$^{-1}$, where S_{Sm}, S_{Ni} and S_O are the stopping cross section of Sm, Ni and O, respectively. N_{Sm}, N_{Ni} and N_O is the atomic percent of Sm, Ni and O, respectively. M_{Sm}, M_{Ni} and M_O is the atomic weight of Sm, Ni and O, respectively. ρ is the density of $SmNiO_3$. The stopping cross sections for all elements are listed in supplementary reference 1.

The instrumental function $F(E, E_i, z)$ is associated to the broadening of the reaction energy window beyond the nuclear reaction resonance width by four physical effects, which independently varies the $^{15}N^{2+}$–H collision energy: (i) $\sigma(E)$: the cross section resonance of the nuclear reaction with the Lorentzian line shape of a Breit–Wigner dispersion function, (ii) $B(E, E_i)$: the energy distribution of the incident ion beam, (iii) $D(E)$: a Doppler effect owning to zero-point vibration of the H atoms within their bonds to the target, and (iv) $S(E, z)$: trajectory length dependent energy straggling of the ^{15}N projectiles in the target material. The instrumental function can be written as [20]:

$$F(E, E_i, z)=\sigma(E)\times B(E, E_i)\times D(E)\times S(E, z) \qquad 1$$

For a specific penetration depth of the ion beam, the spatial resolution of the measured profile is limited by the width of the energy distribution of the ions, and the width of the energy distribution is the full width at half maximum (FWHM) of this distribution. The total FWHM (Γ_T) includes the contribution associate with the width of the resonance itself (Γ_L)， the energy spread of the incoming ion beam (Γ_B), the Doppler broadening (Γ_D), the energy straggling (Γ_S), and the interface inhomogeneity (Γ_I) . It can be written as [1,20]:

$$\Gamma_T = 0.56\Gamma_L + (\Gamma_B^2 + \Gamma_D^2 + \Gamma_S^2)^{1/2} \qquad 2$$

The cross section resonance of the nuclear reaction has the Lorentzian line shape of a Breit–Wigner dispersion function, where E_R (6.385MeV), σ_R, and Γ_L(1.8 keV) denote the resonance energy, the maximum, and the spectral width of the cross section, respectively

$$\sigma(E) = \sigma_R \frac{\Gamma_L^{\,2}/4}{\Gamma_L^{\,2}/4 + (E - E_R)^2} \quad 3$$

The incident ion beam energy distribution (as delivered by the accelerator) can usually be treated approximately by a Gaussian function with a width parameter δ_B (standard deviation), as shown below [2]

$$B(E, E_i) = \frac{1}{\sqrt{2\pi}\delta_B} \exp[-\frac{(E - E_i)^2}{2\delta_B^{\,2}}] \quad 4$$

The experimentally accessible beam energy spread, Γ_B, is the FWHM of this distribution, as show below:

$$\Gamma_B = (8\ln 2)^{1/2}\delta_B \quad 5$$

where the $^{15}N^{2+}$ ion beam at an energy of 6.385 MeV was monochromatized with an energy analyzer of the double-focused magnetic-sector type with an energy dispersion of 2540 mm. Both entrance and exit slit widths of the energy analyzer were carefully measured and set to 3.0 mm.

The spatial confinement of the hydrogen atoms inside their chemical bond potentials causes an inevitable uncertainty of their momentum according to the Heisenberg relation, and the wave function of the resulting zero-point vibration is Gaussian in momentum space (assuming a harmonic oscillator model for the nuclear motion). Although the zero-point vibrational energy of the H nuclei is small (some tens of meV) compared to the nuclear reaction energy E_R, the resulting Doppler shift of the $^{15}N^{2+}$–H collision energy translates into a sizeable Gaussian broadening $D(E)$, as shown below [3]

$$D(E) = \frac{1}{\sqrt{2\pi}\delta_D} \exp(-\frac{E^2}{2\delta_D^{\,2}}) \quad 6$$

The Doppler broadening Γ_D is the FWHM of this distribution, as shown below:

$$\Gamma_D = (8\ln 2)^{1/2}\delta_D \quad 7$$

where the parameter, δ_D, is the width parameter of the Doppler-broadening distribution.

Straggling, an energy fluctuation caused by multiple weak scattering interactions of the ion with the target electrons, simultaneously broadens this energy distribution, as shown in Supplementary Eq. 8. Due to the stochastic nature of the straggling process the energy broadening function is Gaussian and its width parameter increases in proportion to the square root of the ion trajectory length in the analyzed material. Straggling becomes the dominant limitation of the depth resolution from probing depths (z) beyond several nanometer, the precise value depending on the stopping power and straggling cross section of the projectile in the target.

$$S(E,z)=\frac{1}{\sqrt{2\pi}\delta_s(z)}\exp\{-\frac{[E-\Delta E(z)]^2}{2[\delta_s(z)]^2}\} \qquad 8$$

where $\delta_s(z) = (Q_{int.}\Delta h)^{1/2}$, $Q_{int.}$ is the straggling cross section of the interface, $SmNiO_3$, derived from the relationship: $Q_{int.} = Q_{SiO_2}$ (dE/dz)$_{int}$/(dE/dz)$_{SiO_2}$. Here, the stopping power of the interface (dE/dz)$_{int}$ between $SmNiO_3$ and $LaAlO_3$ and approximately equal to be (dE/dz)$_{SNO}$ =3.1945 (keVnm^{-1}). Thus, $Q_{int.}$ = 1.06×10^6×(3.1945/1.8973) eV^2/nm = 1.78×10^6 eV^{2}nm^{-1}, the stopping power of SiO_2 ((dE/dz)$_{SiO_2}$) is equal to 1.8973 keV. The energy loss cross section or stopping powers for is for the compound (e.g., $SmNiO_3$, SiO_2) can be estimated in a first approximation, the so-called Bragg`s Rule, as the stoichiometry weighed average of the respective elemental stopping powers [4,20].

The straggling length, Γ_S, is the FWHM of this distribution is shown as,

$$\Gamma_S=(8\ln 2)^{1/2}\delta_S \qquad 9$$

where the Δh corresponds to the penetrated thickness of ^{15}N ions.

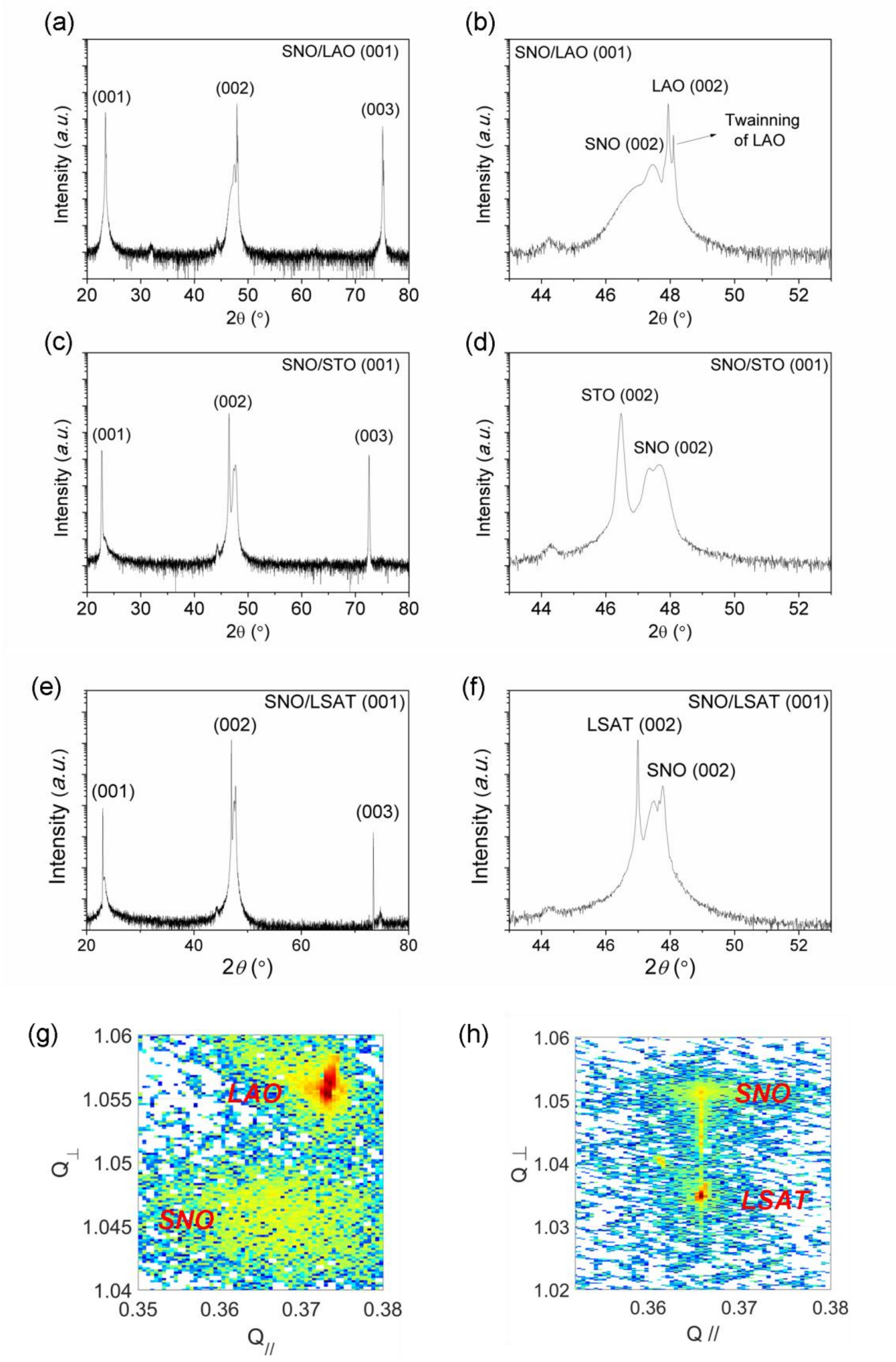

Supplementary Figure 2. Additional XRD and RSM results. XRD patterns (θ-2θ scan) for **(a), (b):** $SmNiO_3/LaAlO_3$ (001) and **(c), (d):** $SmNiO_3/SrTiO_3$ (001) and **(e), (f)** $SmNiO_3$/(La,Sr)(Al,Ta)O_3 (001) samples. Since the diffraction peaks for the film are present beside those for the substrate, the film and substrate show the same crystal structure and orientation, indicating a quasi-single crystalline structure of *as-grown* thin films. Reciprocal space mapping (RSM) of **(g)** $SmNiO_3/LaAlO_3$ (001) and **(h)** $SmNiO_3$/(La,Sr)(Al,Ta)O_3 (001) samples. It can be seen that the $SmNiO_3$ co-lattice grown on the

(La,Sr)(Al,Ta)O_3 (001) substrate, and thereby is tensile distorted owning to the smaller lattice constant of the film material with respective to the substrate. In contrast, the $SmNiO_3$ and $LaAlO_3$ exhibit similar lattice constant, and as-grown $SmNiO_3$ is partially compressive distorted.

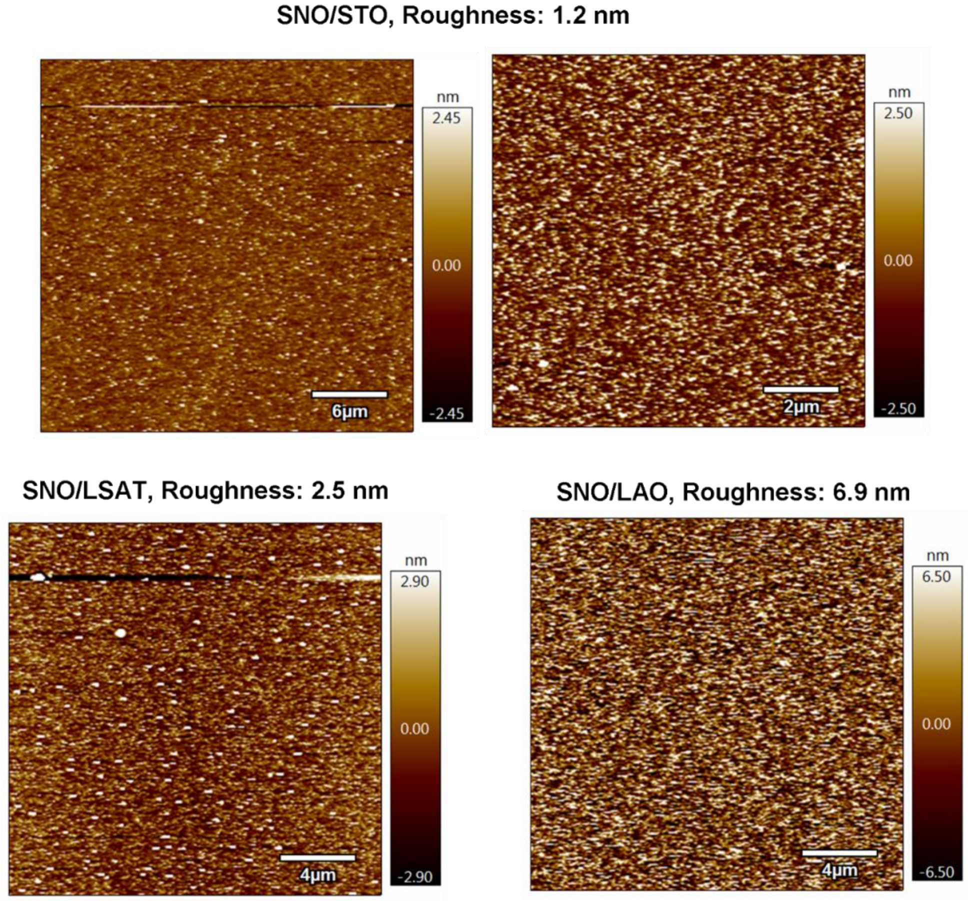

Supplementary Figure 3. Surface morphology results from atomic forth microscopy. Surface morphology for $SmNiO_3$/$SrTiO_3$ (001), $SmNiO_3$/(La,Sr)(Al,Ta)O_3 (001) and $SmNiO_3$/$LaAlO_3$ (001) samples characterized by atomic force microscopy.

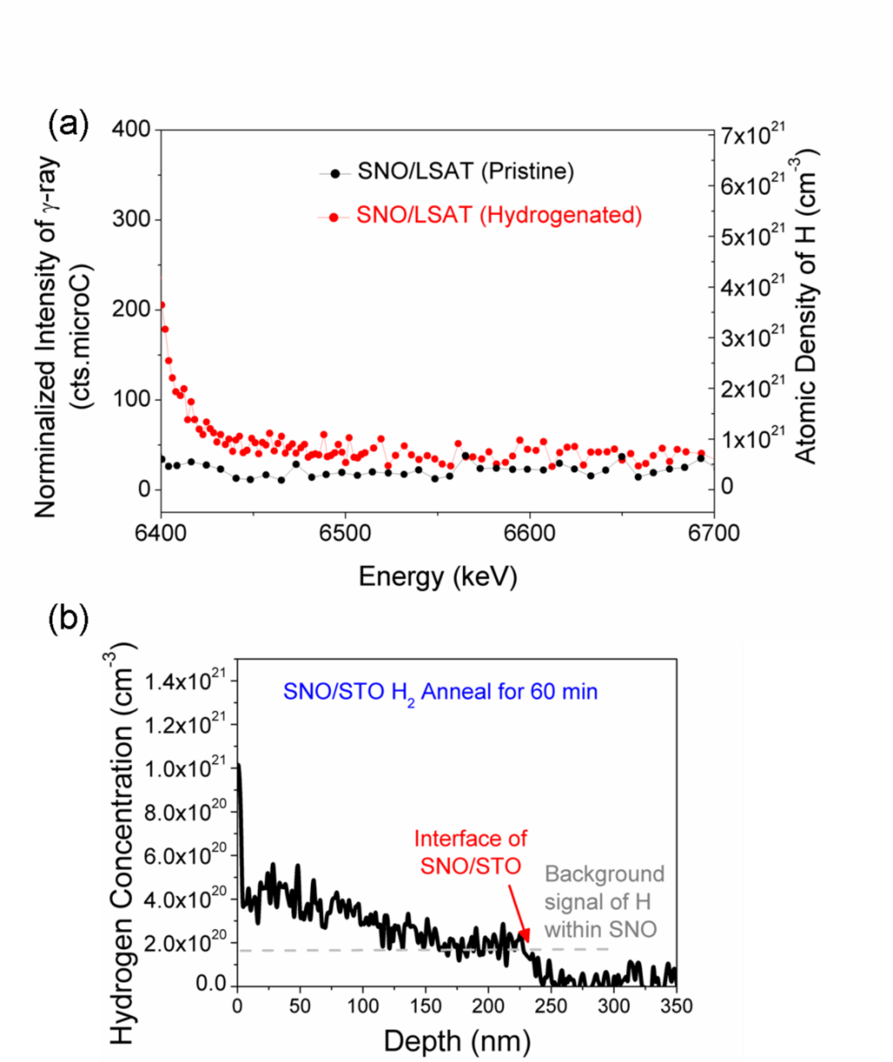

Supplementary Figure 4. Additional results for hydrogen depth profiles. **(a)** Hydrogen (or proton) depth profiles for $SmNiO_3$/(La,Sr)(Al,Ta)O_3 (001) before and after annealing in 1% H_2/He atmosphere for 15 minutes. **(b)** Hydrogen distribution of the hydrogenated (for 60 minute) $SmNiO_3$/(La,Sr)(Al,Ta)O_3 (001) sample from surface to the interface. It is also worth noticing that the pronounced hydrogen concentration minima and maxima close to the surface is attributed to the effects of the resonant nuclear reaction analysis (NRA) instrumental function [20], rather than simply associated to the surface hydrogen concentration. The resonance in the near surface differs from the situation of the bulk, and the near surface peak is resulted from the convolution of the Lorentzian resonance of the $^{1}H(^{15}N,\alpha\gamma)^{12}C$ nuclear reaction at 6.385 MeV [2] with the incident ion beam energy distribution (as delivered by the accelerator)[3] and the Doppler broadening effect induced by the zero-point vibration of the hydrogen atoms against the sample surface[4] and the straggling of the incident ion projectiles inside the sample [5]. The resonant NRA instrumental function is in shape of Voigt profile and the main limitation for the near-surface depth resolution [20], and therefore results in the pronounced concentration minima and maxima close to the surface of the samples.

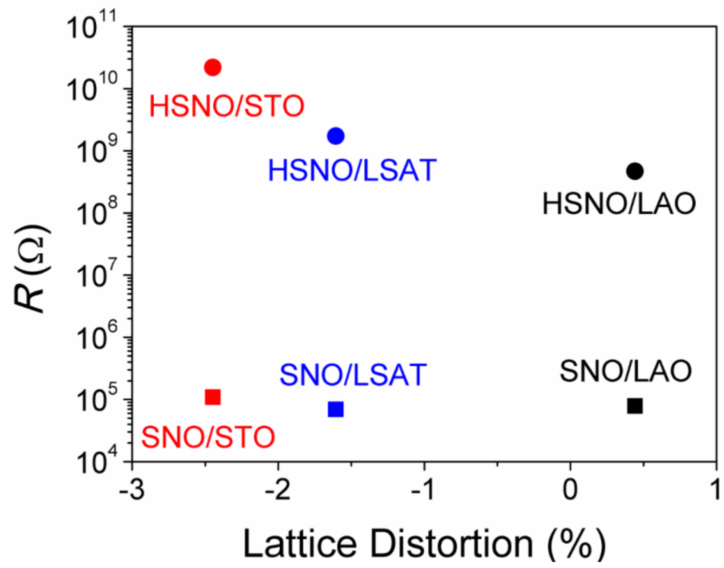

Supplementary Figure 5. The absolute values of R_0, R_H for the films with different strain. It can be seen that before the hydrogenation process, the most tensile strained SNO/STO shows a larger resistance, compared to SNO/LSAT and SNO/LAO. Upon hydrogenation, the difference in resistance was further enhanced. For example, more than 5 orders enhancement in the resistance was observed for SNO/STO, while the increase in resistance for SNO/LSAT is around 4 orders and for SNO/LAO is less than 4 orders.

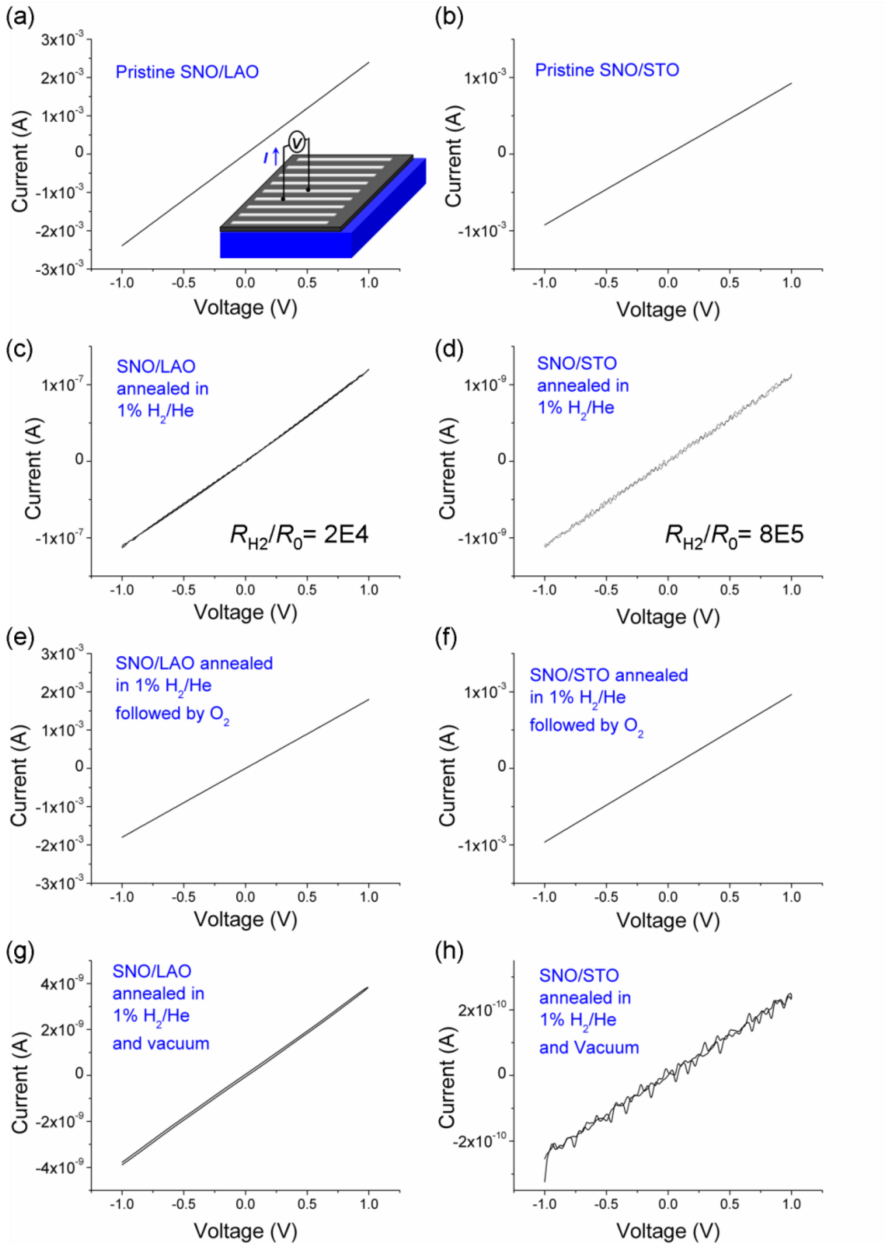

Supplementary Figure 6. Additional current-voltage (*I-V*) characterizations. The *I-V* characterizations of **(a),(b)** the pristine $SmNiO_3/LaAlO_3$ (001) and $SmNiO_3/SrTiO_3$ (001); **(c),(d)** hydrogenated $SmNiO_3/LaAlO_3$ (001) and $SmNiO_3/SrTiO_3$ (001); **(e),(f)** further annealing the hydrogenated $SmNiO_3/LaAlO_3$ (001) and $SmNiO_3/SrTiO_3$ (001) in O_2; **(e),(f)** further annealing the hydrogenated $SmNiO_3/LaAlO_3$ (001) and $SmNiO_3/SrTiO_3$ (001) in vacuum. The hydrogenation results in dramatically increased resistance by 4 and 5 orders for $SmNiO_3/LaAlO_3$ (001) and $SmNiO_3/SrTiO_3$ (001), respectively, while the resistance recovered to the pristine magnitude by dehydrogenation. In contrast, by annealing the hydrogenate $SmNiO_3/LaAlO_3$ (001) and $SmNiO_3/SrTiO_3$ (001) in vacuum at the same temperature and time results in further enhancement in the resistivity.

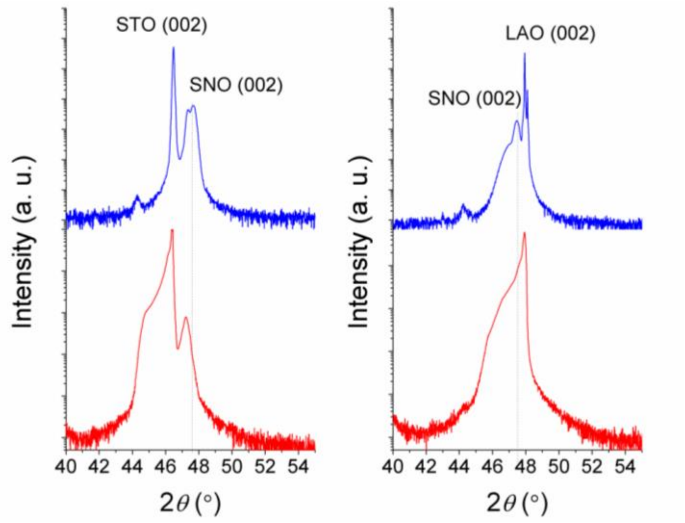

Supplementary Figure 7. Additional X-ray diffraction (XRD) results upon hydrogenation. Comparison of the XRD patterns for $SmNiO_3/SrTiO_3$ (001) and $SmNiO_3/LaAlO_3$ (001) before and after hydrogenation. It can be seen that the hydrogenation reduces the crystalinity of $SmNiO_3$ and shift the diffraction peak towards left, indicating an lattice disordering and expansion after hydrogenation.

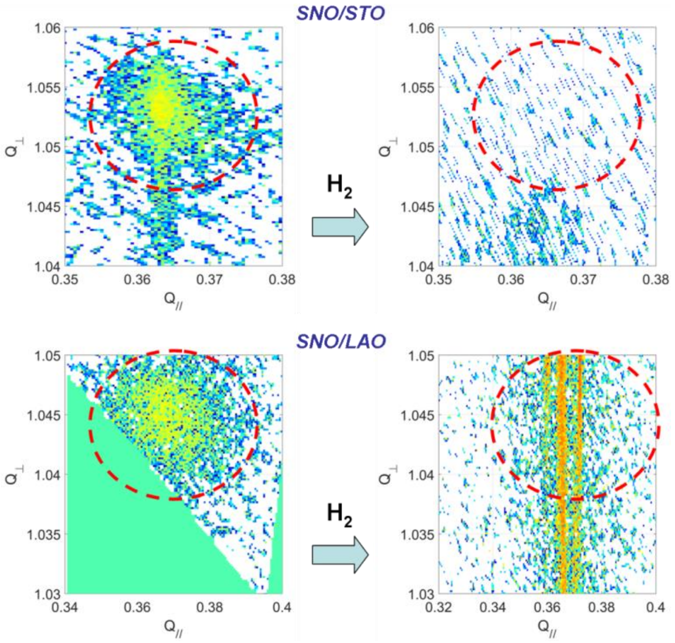

Supplementary Figure 8. Additional Reciprocal space mapping (RSM) results upon hydrogenation. The RSM results of $SmNiO_3/SrTiO_3$ (001) and $SmNiO_3/LaAlO_3$ (001) samples before and after hydrogenation. It clearly demonstrate that the hydrogenation removes the diffraction patterns for the $SmNiO_3$ thin films, which indicates the disordering in lattice at the in-plane as well as cross plane directions. The additional pattern in the RSM of $SmNiO_3/LaAlO_3$ after hydrogenation s at Q// = 0.37 may be caused by the twinning of the LAO substrate.

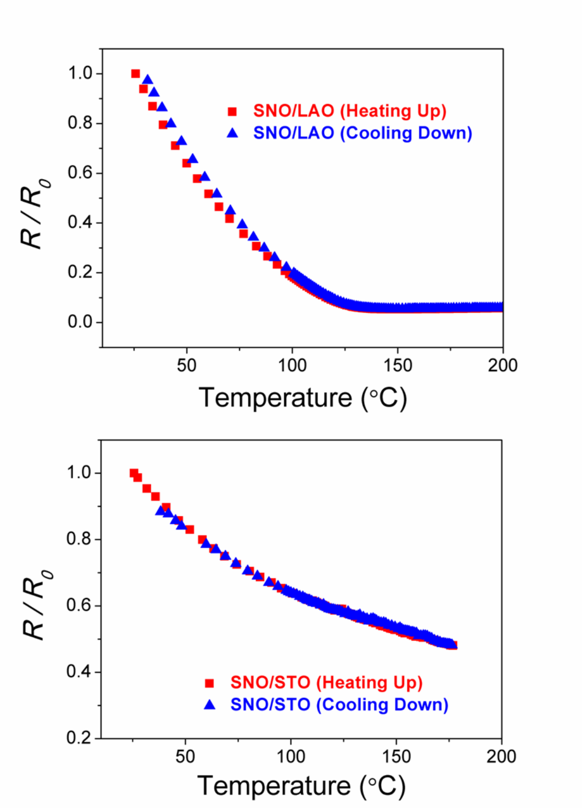

Supplementary Figure 9. Additional results for temperature dependence of resistivity. The variation in resistivity for $SmNiO_3/LaAlO_3$ (001) and $SmNiO_3/SrTiO_3$ (001) are plotted as a function of temperature performed in vacuum.

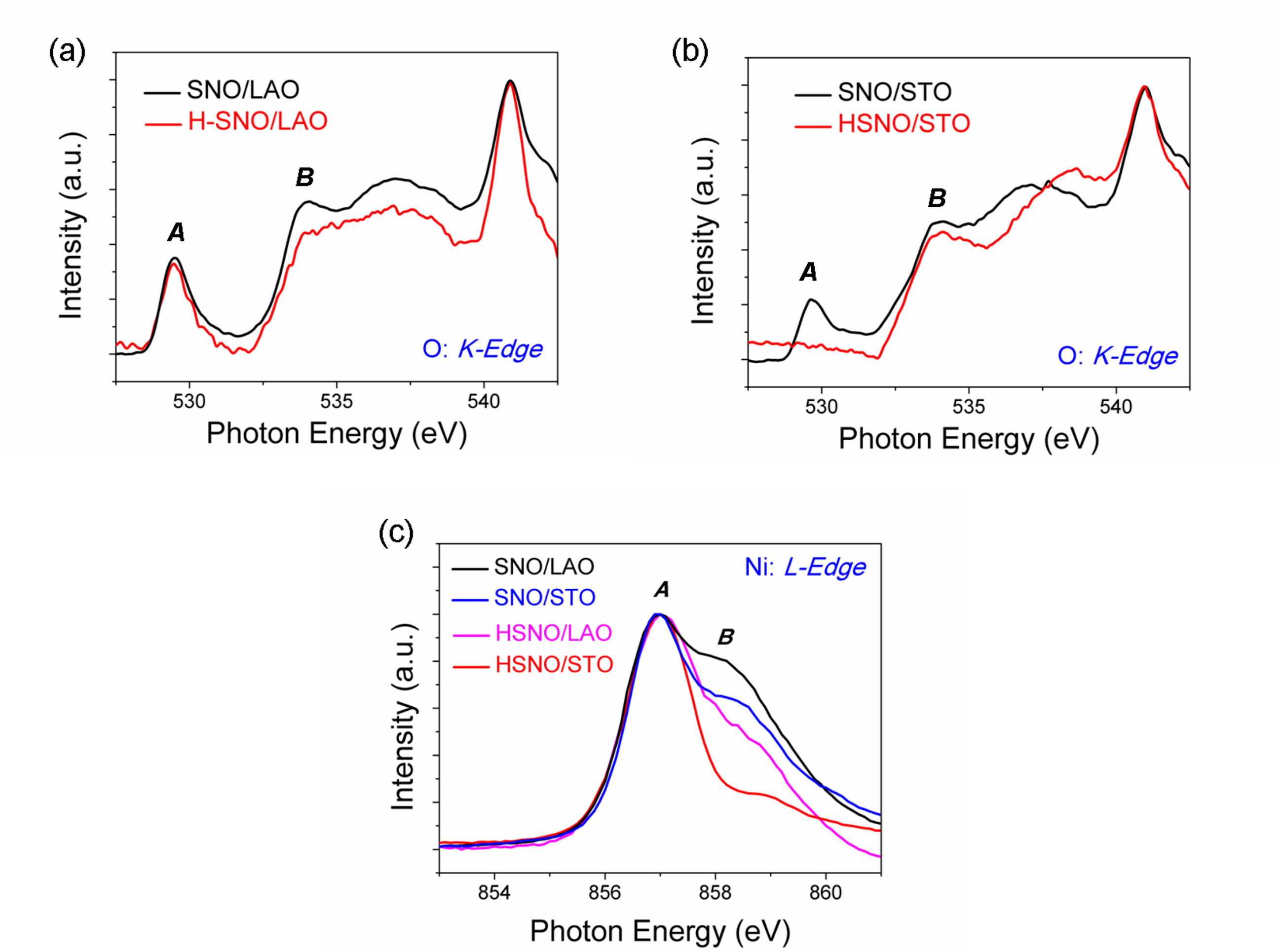

Supplementary Figure 10. Additional near-edge X-ray absorption fine structure (NEXAFS) results. **N**EXAFS results for the O K-edge and Ni L-edge for SNO/LAO and SNO/LAO samples before and after hydrogenation. The pre-peak A in the O: *K* spectrum ～529 eV was attributed to the Ni:3*d*-O:2*p* hybridization (d^8L configuration), while the peaks appearing at the higher photon energies was expected to reflect the more oxygen depleted orbital configurations, i.e., d^9L (Peak B). A more significant reduction in the proportion of pre-peak A is observed in O: *K*-edge for H-SNO/STO compared to H-SNO/LAO upon the hydrogenation process. The Ni: L_3 in the Ni: *L*-edge originates from the Ni 2p→Ni 3d transition, and usually splits into two peaks, reflecting the $t^6_{2g}e^2_g$ (Ni^{2+}, Peak A) and $t^6_{2g}e^1_g$ (Ni^{3+}, Peak B), respectively. The hydrogenation treatment further reduces the proportion in Peak B in Ni: L_3 spectrum more significantly for SNO/STO compared to SNO/LAO.

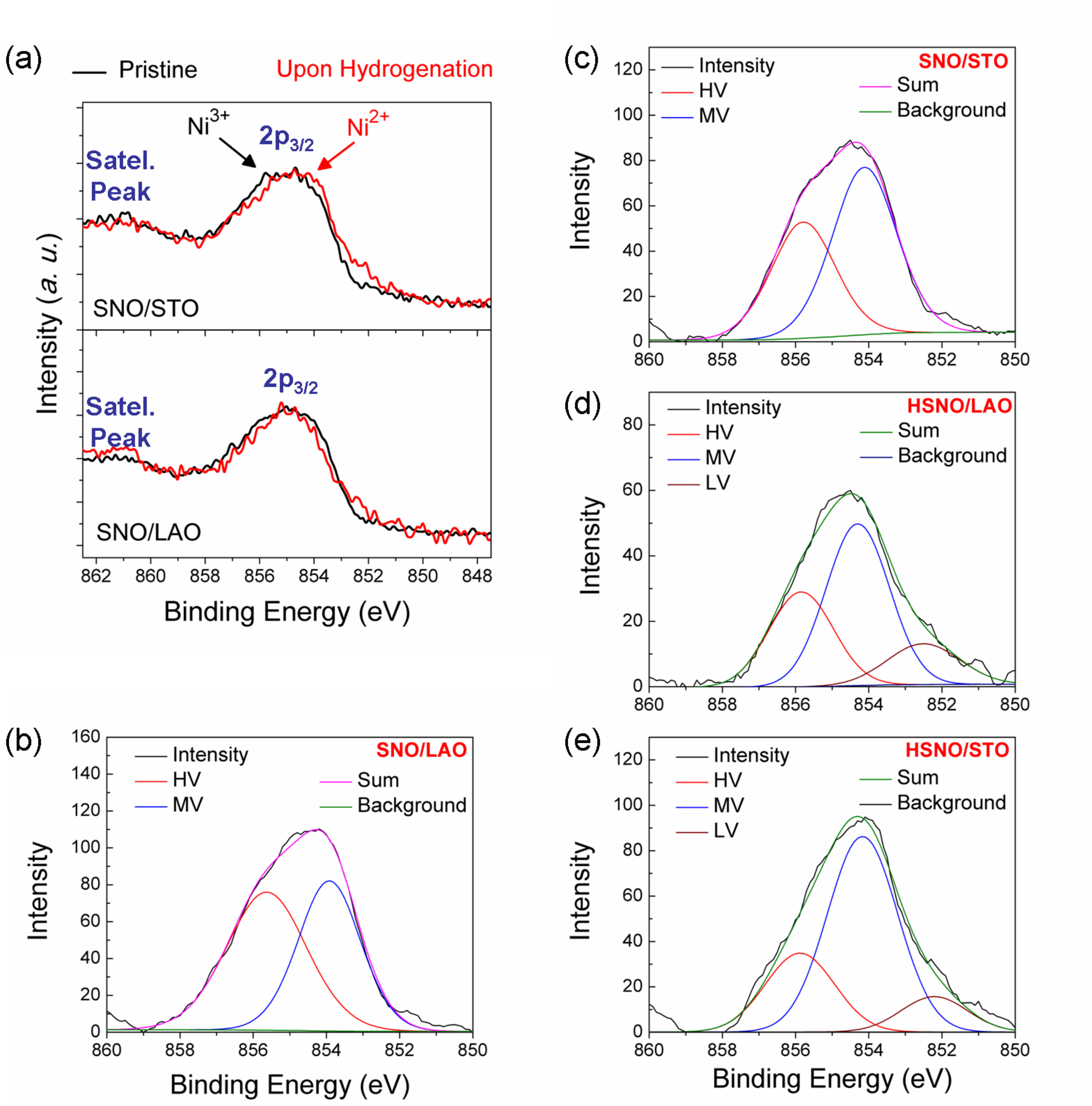

Supplementary Figure 11. Additional XPS results. **(a)** The X-ray photoelectron spectroscopy (XPS) results compared for $SmNiO_3/SrTiO_3$ and $SmNiO_3/LaAlO_3$ before and after the hydrogenation. **(b)-(d)** Curve fitting for the XPS results. It has been pointed out by previous investigations that an accurate quantitative curve fitting of the Ni XPS spectra is nearly not possible, owning to the presence of satellite peaks (sitting 6 eV higher than $2p_{2/3}$) originating form interatomic, non-local electronic coupling and screening effects in Ni^{2+} and Ni^{3+} compounds [6-8]. To demonstrate the more effective reduction in valance state of Ni for SNO/STO as compared to SNO/LAO upon hydrogenation, we utilize the main peak position corresponding to the Ni^{3+} (high valance: HV), Ni^{2+} (middle valance: MV) and Ni^0 (low valance: LV) for the numerical curve fitting, despite their overlapping with the satellite peaks.

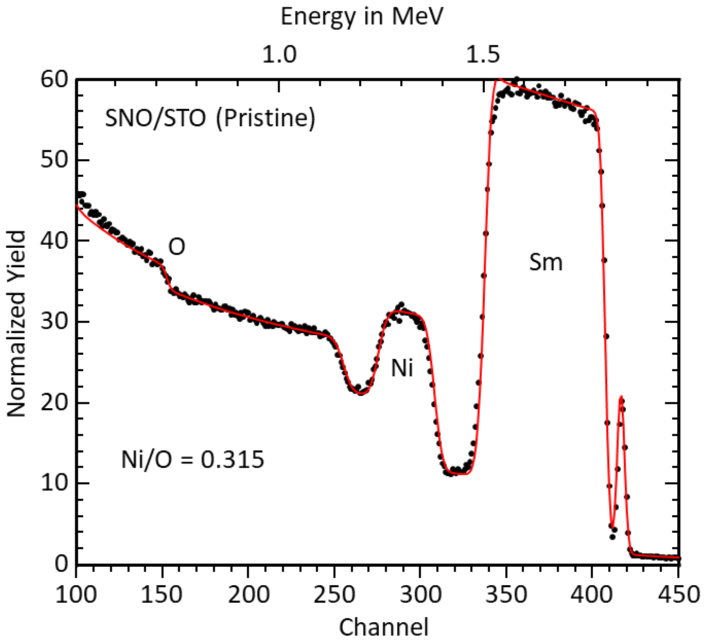

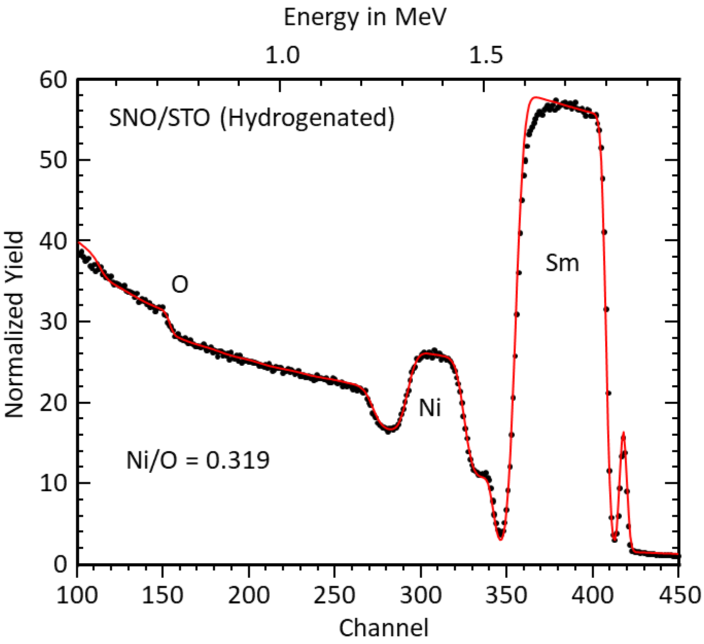

Supplementary Figure 12. Additional Rutherford backscattering (RBS) spectrum. The RBS spectrum for SNO/STO before (upper figure) and after (down figure) the hydrogenation process, demonstrating the variation in the Ni/O ratios before and after the hydrogenation. The black dot is the measured results, while the red line is the fitting curve.

Supplementary Discussions

We have performed first-principles calculations using spin-polarized density functional theory (DFT) based Vienna Ab initio Simulation Package [9]. The electron-ion interaction was described using projector augmented wave (PAW) method [10] The valence electrons configuration for nickel, samarium as well as oxygen are [Ni] $3d^84s^2$, [Sm] $4f^15s^25p^66s^2$ and [O] $2s^22p^4$ respectively. The exchange-correlation between electrons was treated with generalized gradient approximation (GGA) in the Perdew-Burke-Ernzerhof (PBE) form supplementary reference 11 coupled with the Hubbard on-site Columbic correction (GGA + U) [12,13]. The value U_{eff} (Ni) = 2 eV was set to capture the localized state of the 3*d* electrons. An energy cutoff of 500 eV was used for the plane wave basis set. The Brillouin-zone integration was performed within the Monkhorst-Pack scheme [14] using a 6×6×4 *k* mesh for the primitive $SmNiO_3$ unit-cell. In geometry optimization, both shapes and internal structural parameters of pristine unit-cells were fully relaxed until the residual force on each atom is less than 1×10^{-2} eV/Å. The pristine $SmNiO_3$ in our experimental study belongs to the Pnma space group with orthorhombic structure. Varignon et al. demonstrated using GGA+U calculations that there are four possible magnetic configurations, namely, ferromagnetic (FM), T-, E- and S-type anti-ferromagnetic (AFM) ones [15] for Pbnm phase of $SmNiO_3$, with FM being the ground state. Thus, we focus on the FM alignment in the present study. We find that the equilibrium lattice constants are *a*=5.323 Å, *b*=5.486 Å and *c*=7.572 Å, in good agreement with the experiment (5.328 Å, 5.437 Å and 7.568 Å respectively) [16]. The formation energy is −13.64 eV per formula unit. To investigate the role of strain on the diffusion of interstitial H atom (H_i), we studied one H_i atom in a 1×1×1 unit cell of $SmNiO_3$. There are six possible sites for H_i in $SmNiO_3$ [see Supplementary Figure 12a]. Upon relaxation, the H_i is found to bond with the neighboring oxygen anions in all cases, and the most stable site is labeled as G [Supplementary Figure 12].

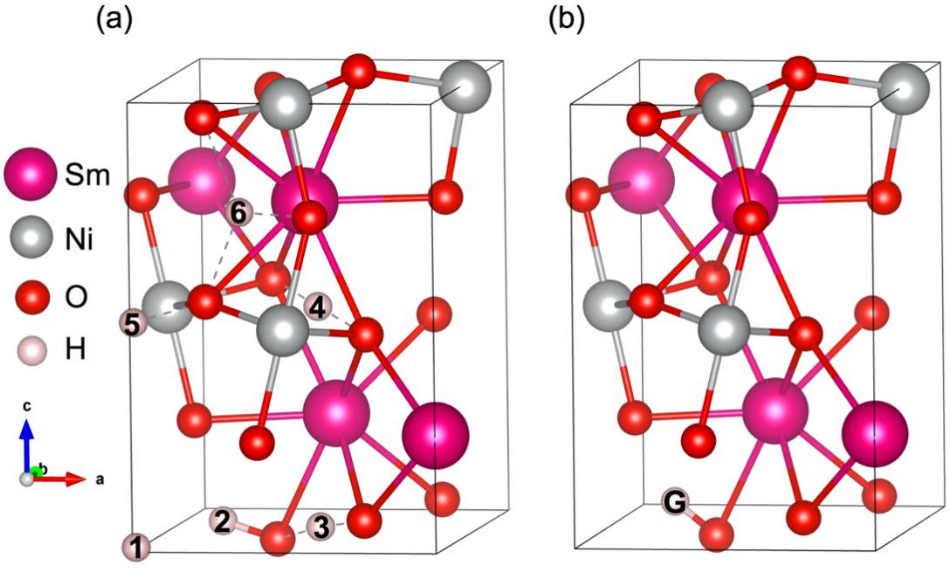

Supplementary Figure 13. The role of strain on the diffusion of interstitial H atom. Ball-and-stick model of **(a)** inequivalent interstitial configurations for H atoms and **(b)** the most energetically favored site in $SmNiO_3$

Supplementary Table 1. Six possible configurations for interstitial H (Hi) and the corresponding equilibrium coordinate and relative stability energy.

Configuration	The relaxed fractional coordinates of H_i	Relative stability energy (eV)
1	(0.000 0.000 0.000)	0.524
2	(0.154 0.785 0.030)	0.000
3	(0.492 0.317 0.025)	0.198
4	(0.500 0.500 0.500)	0.527
5	(-0.017 0.180 0.531)	0.189
6	(0.590 -0.055 0.250)	0.580

To investigate the strain effect on the migration of H_i defect in $SmNiO_3$, we performed climbing image nudged elastic band (CI-NEB) calculations to investigate the energy migration barrier along the [001]

direction under different tension strain [17]. In Supplementary Figs. S13(a) and (b), we present the calculated migration pathway and diffusion barrier of H_i under zero strain. Our calculations show that with the strain increase from 0% to +2%, the energy barrier decreases from 0.56 to 0.49 eV. This implies that the migration of H_i become easy under tension strain.

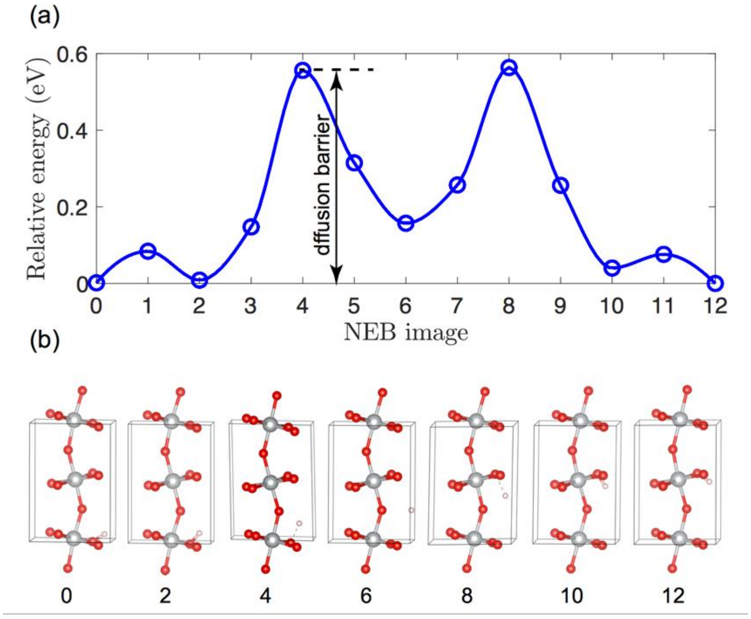

Supplementary Figure 14. The strain effect on the migration of H_i defect in $SmNiO_3$. **(a)** Calculated potential energy surfaces for migration and **(b)** migration pathway for interstitial H atom (the smallest spheres) along [001] direction of the $SmNiO_3$.

Next, we derive the H_i diffusivity from the probability of the H_i defect. Based on the transition state theory,[18] the transition rate for an interstitial defect jumping to one of its neighboring sites is given by Supplementary Eq. 10:

$$\Gamma = \nu_0 e^{-\Delta G/k_{\mathrm{B}}T}, \tag{10}$$

where ν_0 is the attempt frequency, ΔG is the free energy difference between the transition state and the initial state, k_{B} is Boltzmann's constant and T is the absolute temperature. Here we approximate the ΔG by energy barrier between the transition state and the initial state at zero temperature. We note that the contribution from vibrational energy term to the free energy difference is ignored in our current study due to the high computational cost required for the evaluation of the vibrational frequencies of the system in the saddle configuration [19]. With this approximation, we find that at room temperature

the transition rate of H_i defect increases by 15 times under 2% tension strain, compared with the strain-free state.

Supplementary References:

1. Lanford, W. A.; Tesmer, J. R.; Nastasi, M. (Eds.), Handbook of Modern Ion Beam Materials Analysis, Materials Research Society, Pittsburgh, PA (1995), pp. 615-618.

2. Amsel, G.; Maurel, B.; *Nucl. Instrum. Methods Phys. Res.* **1983,** *218*, 183-196.

3. Fukutani, K.; Itoh, A.; Wilde, M.; Matsumoto, M. *Phys. Rev. Lett.* **2002,** *88,* 116101.

4. Wilde, M.; Matsumoto, M.; Fukutani, K.; Liu, Z. Y.; Ando, K.; Kawashima, Y.; Fujieda, S. *J. Appl. Phys.* **2002,** *92,* 4320-4329.

5. Rud, N.; Bøttiger, J.; Jensen,P. S. *Nucl. Instrum. Methods* **1978,** *151,* 247-252

6. Grosvenor, A. P.; Biesinger, M. C.; *Surface Science* **2006,** *600,* 1771-1779

7. J.F. Moulder, W.F.S., P.E. Sobol, K.D. Bomben. Handbook of X-ray photoelectron spectroscopy: a reference book of standard spectra for identification and interpretation of XPS data (1995).

8. van Veenendaal, M. A.; Sawatzky, G. A. *Phys. Rev. Lett.* **1993,** *70,* 2459-2462

9. Kresse G.; Furthmuller, J. *Phys. Rev. B* **1996,** *54,* 11169-11186.

10. G. Kresse and D. Joubert, Phys. Rev. B 59 (1999) 1758-1775.

11. Perdew, J. P.; Burke, K.; Ernzerhof, M. *Phys. Rev. Lett.* **1996,** *77,* 3865-3868.

12. Dudarev, S. L.; Botton, G. A.; Savrasov, S. Y.; Humphreys, C. J.; Sutton, A. P. *Phys. Rev. B* **1998,** *57,* 1505−1509.

13. Anisimov, V. I.; Aryasetiawan, F.; Lichtenstein, A. I. *J. Phys.: Condens. Matter* **1997,** *9*, 767.

14. Monkhorst, H. J.; Pack, J. D. *Phys. Rev. B* **1976,** *13*, 5188–5192.

15. J. Varignon, M. N. Grisolia, J. Íñiguez, A. Barthélémy and M. Bibes, npj Quantum Materials 2 (2017), 21.

16. Ambrosini, A.; Hamet, J. F. *Appl. Phys. Lett.* **2003,** *82*, 727.

17. Henkelman, G.; Uberuaga, B. P.; Jónsson, H. *J. Chem. Phys.* **2000,** *113,* 9901-9904.

18. Eyring, H. The activated complex in chemical reactions. *J. Chem. Phys.* **1935,** *3,* 107–115

19. Wang, Y.; Connétable, D.; Tanguy, D. *Phys. Rev. B* **2015,** *91,* 094106

20. Wilden, M.; Fukutanin, K. Hydrogen detection near surfaces and shallow interfaces with resonant nuclear reaction analysis. *Surface Science Reports* **2014**, 69, 196-295
